# Analysis of the Subculture Effect on the *Auricularia heimuer* Strain ‘HWS1908’ Based on Transcriptome

**DOI:** 10.3390/jof12060437

**Published:** 2026-06-15

**Authors:** Chuang Han, Xiaojia Zhang, Yan Guo, Yinpeng Ma, Shuang Tian, Xiaodong Dai, Piqi Zhang

**Affiliations:** 1 Institute of Microbiology, Heilongjiang Academy of Sciences/National Collection of Edible Fungi (Heilongjiang), Harbin 150010, China17382765294@163.com (S.T.);; 2 Key Laboratory of Agricultural Microbiology of Heilongjiang Province, Northeast Agricultural University, Harbin 150030, China

**Keywords:** *Auricularia heimuer*, ‘HWS1908’, transcriptome, degeneration

## Abstract

The molecular mechanisms of mycelial degeneration during subculturing of *Auricularia heimuer* strain ‘HWS1908’ were investigated across generations G1 to G20. With successive subculturing, mycelial growth rate and compactness declined, cellulase and laccase activities decreased significantly, whereas antioxidant enzyme activities increased. Comparative transcriptome analysis between G1 and G20 identified 2643 differentially expressed genes (DEGs). Gene Ontology (GO) analysis indicated that the DEGs were significantly enriched in terms associated with protein refolding, response to reactive oxygen species, and ferroxidase activity. Kyoto Encyclopedia of Genes and Genomes (KEGG) analysis revealed significant enrichment of DEGs in pathways including phenylpropanoid biosynthesis, peroxisome, fatty acid degradation, and longevity-regulating pathways. Key DEGs, including transcription factors, glycoside hydrolases, lignin-modifying enzymes, chitin synthases, chitinases, ornithine decarboxylase, and Ras/Rho signaling pathway components (*Sos*, *Ras*, *Rac1*), were identified. These genes may be associated with the progressive decline of mycelial vigor, cell wall integrity, and substrate utilization capacity. These findings provide a basis for further exploration of the molecular mechanisms of strain degeneration in *A. heimuer*, and a practical recommendation to limit subcultures to within 20 generations for maintaining high vitality.

## 1. Introduction

*Auricularia heimuer*, a valuable traditional edible and medicinal fungus endemic to China, is not only rich in nutritional components but also possesses various medicinal activities, such as hypoglycemic, antioxidant, and antitussive expectorant effects [1,2]. As the second most widely cultivated edible fungus in China, it is mainly produced in Northeast China, a region with an ecological environment favorable for its growth [3]. Specifically, the *A. heimuer* cultivar ‘HWS1908’ (HMCC No.52700) was recognized as a dominant cultivar in the “2024 Regionalization of High-Quality and High-Efficiency *A. heimuer* Varieties in Heilongjiang Province” and exhibits remarkable adaptability to fruiting under the low-temperature conditions of northern regions [4].

Subculturing is a conventional technique used for the propagation and preservation of fungi [5,6]. However, this approach—particularly during prolonged subculturing and preservation—often induces morphological and physiological alterations, thereby posing a major challenge to the industrial production of edible mushrooms. As a filamentous fungus, *A. heimuer* strains are prone to morphological instability and physiological variation during long-term application, extended storage, and repeated tube subculturing, resulting in substantial economic losses to the industry [7,8,9]. Similar to other crops, strains are a critical factor in edible fungus production, and strain degeneration has been reported in multiple species, including *Flammulina filiformis* [10], *Morchella esculenta* [11], *A. heimuer* [12], *Pleurotus ostreatus* [13], *Cordyceps militaris* [14], and *Volvariella volvacea* [5]. Therefore, it is of considerable significance to clarify the temporal changes in mycelia during subculturing and to elucidate the degeneration mechanism of *A. heimuer*.

Mycelial degeneration is manifested in multiple forms. Morphologically, it is characterized by irregularities on the fruiting body surface and abnormal mycelial growth [15]. With increasing subculture times, the mycelial growth rate and sawdust decomposition capacity of *A. heimuer* gradually decrease [16]. In *C. militaris* strains, degeneration is reflected by mycelial abnormalities or increased branching, with the mycelia becoming curved and irregular in morphology [17]. In addition, increasing the number of subcultures in *V. volvacea* results in reduced mycelial biomass and growth rate [5]. Although mycelial abnormalities are present, they are often difficult to detect or directly associate with yield losses; notably, the morphological differences between degenerated and normal strains usually become apparent only at the fruiting body stage, when yield losses are already irreversible [18].

In basidiomycete fungi, repeated subculture-induced strain deterioration arises from oxidative injury that drives cellular senescence, a physiological aging process accompanied by disturbed metabolism across multiple filamentous edible fungi [19]. However, the critical subculture threshold at which these damages become irreversible and the underlying transcriptional program remains poorly defined in *A. heimuer*. Therefore, further research is required to determine the subculture generation threshold for *A. heimuer* mycelial propagation and to investigate the molecular mechanisms involved in strain degeneration using transcriptome sequencing.

Unlike traditional phenotypic and physiological observations that only reflect macroscopic degenerative symptoms, transcriptome analysis allows us to unravel the complex network of underlying gene interactions, offering a molecular-level, spatiotemporal resolution of the cellular events that precede the macroscopic mycelial degeneration caused by continuous subculture. We hypothesize that the continuous subculture of *A. heimuer* induces progressive mycelial phenotypic degeneration, which is mainly driven by dysregulated antioxidant metabolism and lignocellulolytic enzyme synthesis at the transcriptional level, and the degenerative phenotypes will become more significant with the increase in subculture generations. Previous studies demonstrated that 10 consecutive subcultures caused distinct phenotypic changes in *A. heimuer* mycelia [20]. To further intensify these degenerative changes, the 10th-generation (G10) mycelia of the *A. heimuer* cultivar ‘HWS1908’ were continuously subcultured to the 20th generation (G20), and transcriptome analysis was performed between G1 and G20 to clarify the molecular mechanisms driving strain degeneration in *A. heimuer*. Furthermore, this study not only helps reduce the substantial economic losses caused by strain degeneration in large-scale cultivation, but also provides the molecular basis necessary to establish standardized subculture and quality-control protocols for China’s domestic edible mushroom industry. By further investigating the mechanism of strain degeneration, the understanding of genetic variation patterns in *A. heimuer* strains can be improved, thereby providing a theoretical basis for strain selection.

## 2. Materials and Methods

### 2.1. Strains and Culture Conditions

*A. heimuer* ‘HWS1908’ (HMCC No. 52700) was provided by the National Collection of Edible Fungi (Harbin, Heilongjiang, China). All subculturing and inoculation procedures were performed under sterile conditions using a laminar flow hood to prevent contamination. The strain was cultivated on potato dextrose agar (PDA, 4.9% *w*/*v*, natural pH) in a controlled environment chamber at 23 ± 1 °C with a relative humidity of 60 ± 5% in the dark.

### 2.2. Acquisition of the Secondary Strain

The fruiting body of strain ‘HWS1908’ was subjected to tissue isolation to obtain the mother strain, which was subsequently inoculated onto a PDA plate medium and designated as mycelium G0. Mycelial plugs (6 mm diameter) were taken from the leading edge (actively growing peripheral zone) of the colony and transferred to the center of fresh PDA plates. This procedure was repeated every 15 days (or when the mycelium completely covered the 90 mm Petri dish) to obtain successive generations from G1 to G20. Six independent replicate plates were maintained for each generation.

### 2.3. Observation of Mycelial Morphology

Macroscopic observation: Strains of G1~G20 were cultured on PDA. Colony morphology was photographed at fixed 5 d, 10 d and 15 d post-inoculation. These three time points cover the whole developmental cycle including initial germination, rapid expansion and mature stationary phase. Colony diameters were measured continuously until mycelia fully covered the plates [21].

Growth rate (mm/d) = Linear growth amount in the culture dish (mm)/Growth days (d)

Microscopic observation: Two glass slides were inserted 2 cm away from the inoculation point. During observation, the glass slides at the edge of the culture medium were removed, and mycelial morphology was observed using an Olympus BX53F (Olympus Corporation, Tokyo, Japan) optical microscope at 40× magnification [20].

### 2.4. Antioxidant Enzyme Activity Determination

Using a 5 mm diameter circular puncher, bacterial colonies grown on Petri dishes of the passage strains G1, G8, G16, and G20 were collected and inoculated into potato dextrose broth (PDB, 2.6% *w*/*v*, natural pH) for 7 days at 25 °C in a shaking incubator at 160 r/min. After liquid cultivation, the samples were centrifuged at 12,000 r/min for 10 min at 4 °C. The supernatant was discarded, and the precipitated mycelium was gently blotted with filter paper to remove excess surface moisture. 0.5 g of the blotted wet mycelium was collected from each sample. The activity levels of antioxidant enzymes, including superoxide dismutase (SOD), peroxidase (POD), and catalase (CAT), were determined using reagent kits provided by Suzhou Greister Biotechnology Co., Ltd. (Suzhou, China) [22]. SOD: Determined using the nitroblue tetrazolium (NBT) photoreduction method. One unit (1 U) of enzyme activity was defined as the inhibition of NBT photoreduction by 50%. POD: Determined using the crotonic acid method. One unit (1 U) of enzyme activity was defined as a 0.01 change in absorbance per minute. CAT: Determined using the ultraviolet absorption method. One unit (1 U) of enzyme activity was defined as a 0.01 decrease in absorbance per minute. Each group of samples contained three replicates.

### 2.5. Extracellular Degrading Enzyme Activity Determination

The samples were centrifuged at 12,000 r/min for 10 min at 4 °C, and the supernatant was collected as the crude enzyme solution. Extracellular degrading enzyme activities were determined using kits from Beijing Boxbio Science & Technology Co., Ltd., Beijing, China [23]. Laccase: The ABTS method was used for determination. One unit (1 U) of enzyme activity was defined as the amount of enzyme required to oxidize 1 nmol of ABTS per minute in every mL of liquid sample. Cellulase: The filter paper method was used for determination. One unit (1 U) of enzyme activity was defined as the catalytic production of 1 μg of glucose per minute for each milliliter of liquid sample. Hemicellulase: The xylan method was used for determination. One unit (1 U) of enzyme activity was defined as the amount of enzyme required to decompose xylan into 1 μmol of reducing sugar per minute at 50 °C and pH 6.0 for every milliliter of liquid sample. Each group of samples contained three replicates.

### 2.6. Transcriptome Sequencing and Analysis

Using the G1 and G20 mycelia of *A. heimuer* as samples, 100 mg of each sample was accurately weighed. Each group contained three biological replicates, resulting in a total of six samples for transcriptome sequencing. All samples were immediately snap-frozen in liquid nitrogen and subsequently transported to Sangon Biotech (Shanghai) Co., Shanghai, Ltd. [24] on dry ice for total RNA extraction. RNA integrity was evaluated by electrophoresis on a 1% agarose gel; all samples used in this study displayed clear rRNA bands without evidence of degradation. After RNA quality was verified to be qualified, complementary DNA (cDNA) libraries were constructed. Following the qualification of the constructed libraries, high-throughput paired-end sequencing (2 × 150 bp) was performed on the Illumina HiSeq 2500 platform (Illumina, Inc., San Diego, CA, USA), with a minimum of 38 million reads per sample.

The reference genome used in this study was *A. heimuer* version GCA_050574805.1 (JBNRRR000000000, SAMN 44541401) [25]. The genome index was constructed using HISAT2 software (version 2.1.0), and the obtained clean reads were aligned to the reference genome. Differentially expressed genes (DEGs) between treatment groups were identified using DESeq2 v1.22.1 software, with screening criteria of q-Value < 0.05 and |log_2_FoldChange| > 1.0. Subsequently, functional enrichment analyses of DEGs were conducted, including Carbohydrate-Active enZYmes (CAZy) annotation, Gene Ontology (GO) functional enrichment analysis, and Kyoto Encyclopedia of Genes and Genomes (KEGG) metabolic pathway analysis, in order to clarify the biological functions of the DEGs.

### 2.7. qRT-PCR Verify

The qRT-PCR experiment was conducted according to previously reported methods [26]. The adenine phosphoribosyltransferase (*APRT*) gene was selected as the internal control, and this gene has been verified to be stably expressed for qPCR normalization across different developmental stages of fungi in previous research [27]. Four candidate genes used for RT-qPCR validation were randomly screened from all annotated differentially expressed genes derived from RNA-Seq data, with no preselection based on gene functional classification. Relative expression levels were calculated using the 2^–ΔΔCt^ method. All primer sequences are listed in Table S1.

### 2.8. Statistical Analysis

The results were analyzed using IBM SPSS Statistics 24 through one-way ANOVA followed by Duncan’s multiple-range test (*p* ≤ 0.05). GraphPad Prism software (version 10.4.1) and Adobe Illustrator 2020 were used for graph and figure preparation, respectively. The data are presented as means ± standard deviations.

## 3. Results

### 3.1. Morphological Differences

**Figure 1:**
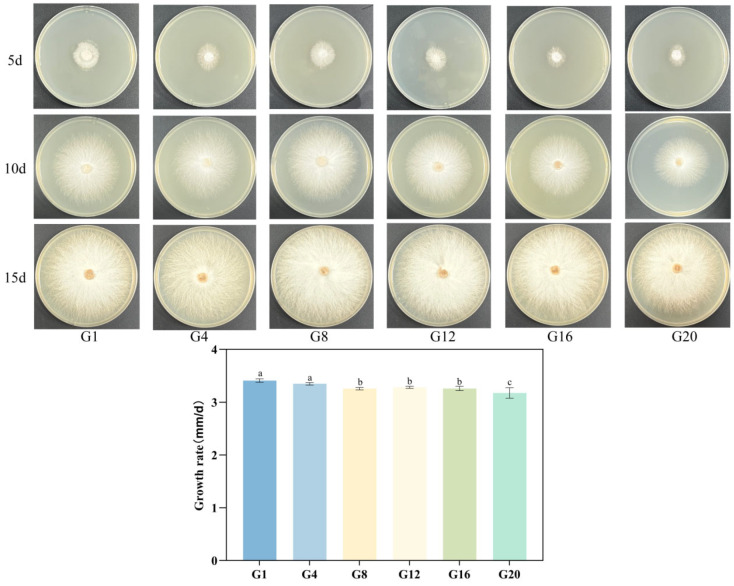
The strain HWS1908 subculture mycelial morphology and growth rate of different generations. Different lowercase letters indicate significant differences (*p* < 0.05).

Consistent with earlier reports [20], gradual alterations in the mycelial morphology of strain HWS1908 were observed during prolonged subculturing. From the 1st to the 20th generation (G1–G20), the mycelia remained white and floccose, and no clear macroscopic differences in colony color or surface texture were detected among the different generations (Figure 1). Nevertheless, relative to G1, strains from G8 to G20 showed obvious reductions in mycelial germination rate and growth vitality, together with decreased mycelial compactness and a pronounced reduction in growth rate. At G20, the growth rate declined to 3.17 ± 0.08 mm/d, which was significantly lower than that recorded for G1 (3.41 ± 0.03 mm/d, *p* < 0.05).

### 3.2. Changes in Antioxidant Enzyme Activities

**Figure 2:**
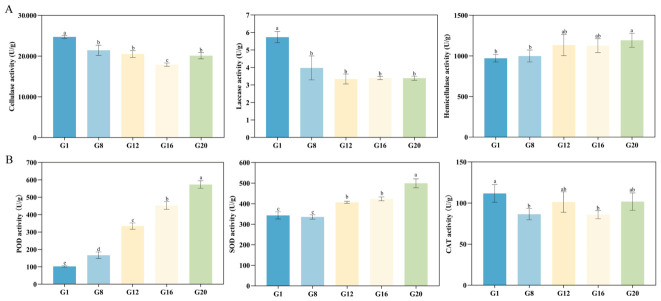
Antioxidant and extracellular degrading enzyme activities of subcultured strains. (**A**) Antioxidant enzyme; (**B**) Extracellular degrading enzyme. Different lowercase letters indicate significant differences (*p* < 0.05).

To evaluate the effects of long-term subculturing on the antioxidant system of *A. heimuer* strain HWS1908, the activities of superoxide dismutase (SOD), peroxidase (POD), and catalase (CAT) were measured in mycelia from different generations (G1–G20). The activities of all three enzymes changed significantly with increasing subculture generations (Figure 2). As core enzymes of the antioxidant system, POD and SOD activities showed a significant increasing trend with successive subculturing (*p* < 0.05), reaching peak levels at G20. CAT activity exhibited a transient decrease at G8 but subsequently increased gradually, showing an overall upward trend, although its response to long-term subculture stress differed from that of POD and SOD. These results indicate that long-term subculturing induces activation of the antioxidant enzyme system in *A. heimuer* mycelia.

### 3.3. Changes in Extracellular Degrading Enzyme Activities

During long-term subculture, the extracellular degrading enzyme activities of strain HWS1908 exhibited distinct differential variation patterns. Cellulase activity decreased significantly from G8 onward and reached the lowest level at G16, with a slight recovery at G20, although it remained markedly lower than that of the initial generation. Laccase activity was highly sensitive to subculture stress; its activity decreased by 30.69% at G8 compared with G1 and continuously declined with increasing subculture generations, reaching only 3.84 ± 0.09 U/g at G20. Hemicellulase activity remained stable without obvious fluctuation from G1 to G16, whereas it increased significantly by 22.73% at G20 relative to G1. The results indicate that long-term continuous subculture causes a progressive decline in the activities of key enzymes responsible for cellulose and lignin degradation in *A. heimuer*.

### 3.4. Analysis of Transcriptome Sequencing Data

**Figure 3:**
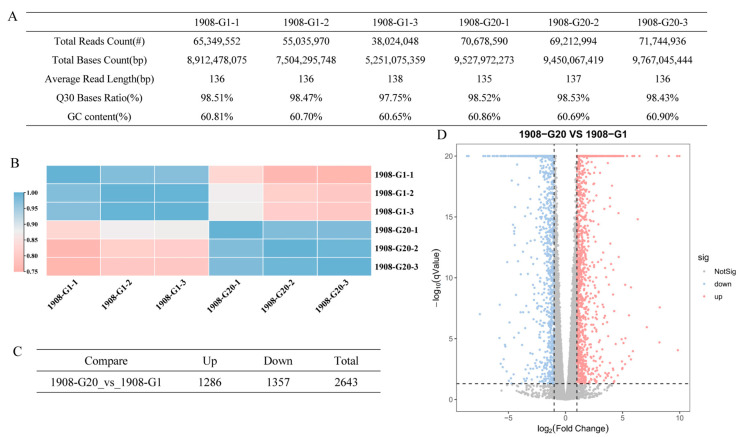
Analysis of transcriptome sequencing data. (**A**) RNA sequencing data statistics table; (**B**) Heat map of the expression level correlation of the samples; (**C**) Statistics table of differentially expressed genes; (**D**): Volcano plot of differentially expressed genes.

For all subcultured strains (G1 and G20), the Q30 ratio of clean bases exceeded 97.35%, and the GC content remained stable within the range of 60.80–60.97%. These results confirmed high-quality sequencing data with excellent biological reproducibility (Figure 3A). Pearson correlation analysis of gene expression revealed that the correlation coefficients within each group were close to 1.0, demonstrating satisfactory intra-group repeatability. In contrast, distinct expression patterns were observed between the G1 and G20 groups, implying that continuous subculture induced extensive and systematic transcriptomic alterations in the mycelia (Figure 3B).

Using screening thresholds of q value < 0.05 and |log_2_FoldChange| > 1.0, a total of 2643 differentially expressed genes (DEGs) were identified, among which 1286 genes were significantly upregulated and 1357 were downregulated (Figure 3C). The volcano plot further visualized the overall distribution of these DEGs, which were widely dispersed on both sides of the plot. Collectively, these DEGs are speculated to exert important regulatory effects on the progressive degenerative phenotype of strains during long-term subculture (Figure 3D).

### 3.5. GO Annotation Analysis of DEGs

To further explore the functional differences in differentially expressed genes (DEGs) between the two comparison groups, Gene Ontology (GO) annotation, classification, and enrichment analyses were performed on the identified DEGs. A total of 520 DEGs were successfully annotated and enriched in diverse GO terms. According to q-Value ranking, the top 30 significantly enriched terms included 21 terms assigned to biological process (BP), 7 terms to cellular component (CC), and 2 terms to molecular function (MF) (Table S2).

**Figure 4:**
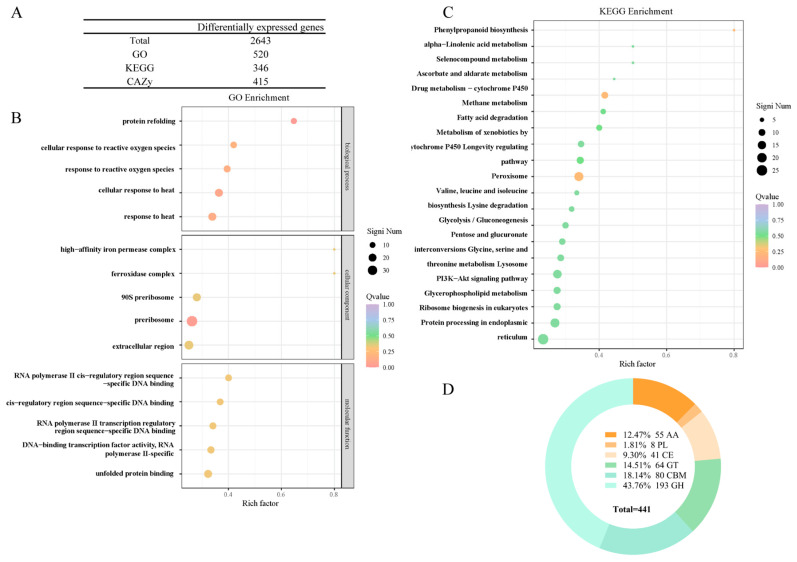
Overview of functional enrichment of DEGs from *A. heimuer* 1908-G20 vs. 1908-G1. (**A**) Summary statistics of DEGs and their annotations in the GO, KEGG, and CAZy databases. (**B**) GO enrichment analysis of DEGs. The top five significantly enriched terms (ranked by q-value) are shown for each of the three GO categories: BP, CC, and MF. (**C**) KEGG enrichment analysis of DEGs. The top 20 significantly enriched pathways (ranked by q-value) are presented. (**D**) Proportions of differentially expressed genes in each CAZy family (AA, PL, CE, GT, CBM, GH).

The top five enriched terms in the BP, CC, and MF categories were further summarized, respectively (Figure 4B). The dominant BP terms included protein refolding (GO:0042026), cellular response to heat (GO:0034605), response to heat (GO:0009408), response to reactive oxygen species (GO:0000302), cellular response to reactive oxygen species (GO:0034614), and rRNA processing (GO:0006364). The top five CC terms included preribosome (GO:0030684), 90S preribosome (GO:0030686), extracellular region (GO:0005576), high-affinity iron permease complex (GO:0033573), and ferroxidase complex (GO:1905862). For the MF category, the top five terms comprised ferroxidase activity (GO:0004322), oxidoreductase activity acting on metal ions with oxygen as the acceptor (GO:0016724), DNA-binding transcription factor activity (GO:0003700), snoRNA binding (GO:0030515), and DNA-binding transcription repressor activity (GO:0001217).

### 3.6. KEGG Annotation Analysis of DEGs

KEGG enrichment analysis demonstrated that the differentially expressed genes (DEGs) were significantly enriched in multiple pathways related to metabolism, redox regulation, stress response, and signal transduction (Figure 4C). The major enriched pathways included phenylpropanoid biosynthesis (ko00940), peroxisome (ko04146), fatty acid degradation (ko00071), longevity regulating pathway (ko04211), methane metabolism (ko00680), alpha-linolenic acid metabolism (ko00592), selenocompound metabolism (ko00450), ascorbate and aldarate metabolism (ko00530), as well as cytochrome P450-associated pathways, including drug metabolism-cytochrome P450 (ko00982) and metabolism of xenobiotics by cytochrome P450 (ko00980). Several fundamental carbohydrate and amino acid metabolic pathways were also significantly enriched, such as glycolysis/gluconeogenesis (ko00010), pentose and glucuronate interconversions (ko00040), glycine, serine and threonine metabolism (ko00260), lysine degradation (ko00310), and valine, leucine and isoleucine biosynthesis (ko00290).

In addition, a number of pathways involved in cellular structure maintenance, material transport, and aging-related regulation were enriched, including glycerophospholipid metabolism (ko00564), lysosome (ko04142), PI3K-Akt signaling pathway (ko04151), and eukaryotic ribosome biogenesis (ko03008). Meanwhile, protein processing in the endoplasmic reticulum (ko04141) was also enriched among the DEGs. Among these, the peroxisome pathway (ko04146) and the phenylpropanoid biosynthesis pathway (ko00940) emerged as the most significantly enriched “winning” pathways, highlighting that oxidative stress and lignin metabolism disruption are central to the degeneration process. Notably, the enrichment of the peroxisome pathway—coupled with the upregulation of antioxidant enzyme activities (Section 3.2) and GO terms such as “response to reactive oxygen species” (Section 3.5)—suggests that oxidative stress acts as the primary trigger, while metabolic dysfunction (e.g., fatty acid degradation and carbohydrate metabolism) is a downstream consequence. Thus, the causative cascade is likely that oxidative stress initiates metabolic failure, rather than metabolic abnormalities secondarily driving oxidative damage. Collectively, long-term subculture triggered extensive disturbances in aromatic compound metabolism, lignin degradation, redox homeostasis, energy metabolism, and cellular senescence signaling in *A. heimuer*, ultimately disrupting intracellular metabolic balance.

### 3.7. CAZy Annotation Analysis of DEGs

**Figure 5:**
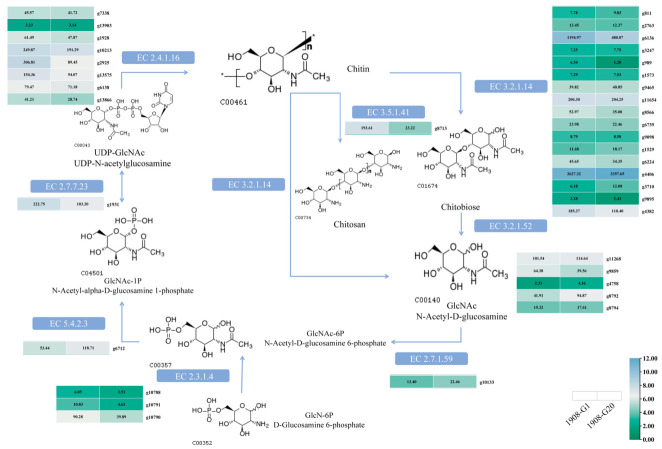
Analysis of amino sugar and nucleotide sugar metabolism pathways. The asterisks (*) indicate the connection points of the repeating units.

Carbohydrate-active enzyme classification was completed based on the CAZy database into six major functional classes, including AA (*n* = 59), GH (*n* = 193), GT (*n* = 64), CBM (*n* = 80), CE (*n* = 41), and PL (*n* = 8), each of which was further classified into several enzyme families. These enzymes are mainly associated with the generation of organic acids by microbial strains. GH was dominant, accounting for 43.76% of the total CAZy genes (Figure 4D). The predominance of GH family genes reflects the wood-rotting lifestyle of *A. heimuer*, whose genome contains a more abundant repertoire of glycoside hydrolase genes than other mushrooms [28]. GH18 chitinases are involved in a broad range of physiological processes, including tissue degradation and remodeling, as well as nutrient acquisition [29]. In the present study, a total of 17 GH18 family genes were identified among the DEGs. Among them, 10 genes were significantly downregulated. Notably, three downregulated transcripts (g9895, g989, and g6136) were annotated as chitinases, all of which exhibited markedly low expression levels (Figure 5; Table S3).

Multiple CAZyme families involved in wood degradation have been reported previously [30]. In this study, a total of 15 such families comprising 83 differentially expressed genes (DEGs) were annotated, among which 51 genes were significantly downregulated (Table S3). GH6 enzymes specifically cleave β-1,4-glycosidic bonds in cellulose and β-1,4-glucans [31]. Consistent with the decreased cellulase activity shown in Figure 2A and Table S4, GH6-encoding genes were markedly downregulated in 1908-G20 relative to 1908-G1. Lignin is a complex aromatic polymer composed of phenylpropanoid structural units [32]. The AA2 family consists of secreted class II lignin-modifying heme peroxidases, mainly including manganese peroxidase (MnP), lignin peroxidase (LiP), and versatile peroxidase (VP) [33,34]. In the present study, all four DEGs assigned to the AA2 family were significantly downregulated in 1908-G20 compared with 1908-G1. The AA6 family encodes intracellular 1,4-benzoquinone reductases, which contribute to the biodegradation of aromatic compounds through regulation of quinone intermediates [35]. The DEGs of 1908-G20 vs. 1908-G1 were enriched in four genes belonging to the AA6 family, and all of them were downregulated. Taken together, integrated transcriptomic and enzymatic analyses reveal that the coordinated downregulation of CAZyme families involved in lignocellulose degradation directly explains the gradual reduction in extracellular cellulase and laccase activities observed in Section 3.3. These findings reflect impaired substrate utilization capacity during continuous subculture and mycelial degeneration of *A. heimuer* (Table S4).

### 3.8. Analysis of Amino Sugar and Nucleotide Sugar Metabolism Pathways

In the fungal strain 1908-G20, the cell wall is mainly composed of chitin and β-glucan [36]. UDP-*N*-acetylglucosamine (UDP-GlcNAc) is the direct precursor for chitin synthesis, and its homeostasis is critical for maintaining cell wall integrity [37]. All eight genes encoding chitin synthase (EC 2.4.1.16), which polymerizes UDP-GlcNAc into chitin, showed a downward trend in expression (TPM). In addition, the upstream key enzyme UDP-*N*-acetylglucosamine diphosphorylase (EC 2.7.7.23), which is responsible for UDP-GlcNAc synthesis, was also downregulated (gene g1931, log_2_FC = −1.20). These findings indicate that both precursor supply and polymerization capacity for chitin synthesis are weakened in 1908-G20.

Chitinase (EC 3.2.1.14) degrades chitin by cleaving β-(1→4)-glycosidic linkages [38]. During the initial stage of chitin degradation, a core highly expressed chitinase gene, g6136, was significantly downregulated by 59.76% (log_2_FC = −1.40). Another chitinase gene, g4406, remained highly expressed and was upregulated by 27.80%. This differential expression pattern within the chitinase family suggests that the overall chitinolytic activity of strain 1908-G20 was not completely impaired. Furthermore, gene g8731 encoding chitin deacetylase (EC 3.5.1.41) also exhibited a downregulation trend (log_2_FC = −3.14). This finding implies that the conversion of chitin to chitosan may be reduced, although the statistical evidence remains marginal. Taken together, although chitin synthesis is attenuated, a partially active degradation system is still maintained—suggesting that the fungus retains a compensatory capacity for cell wall homeostasis, which may delay but not prevent the progression of degeneration.

### 3.9. Analysis of Regulation of Actin Cytoskeleton

**Figure 6:**
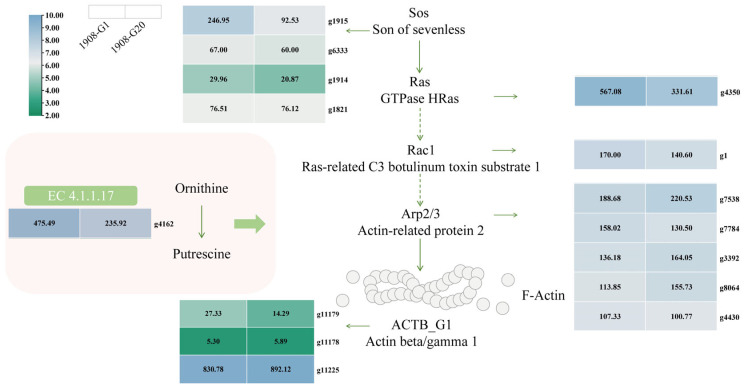
Analysis of the regulation of the actin cytoskeleton.

Polyamines, including putrescine, spermidine, and spermine, are essential for fungal hyphal growth, stress responses, and aging regulation, and their intracellular levels decline markedly with increasing hyphal age [39]. Ornithine decarboxylase (ODC, EC 4.1.1.17) is the rate-limiting enzyme catalyzing the conversion of ornithine to putrescine during polyamine biosynthesis [40]. Transcriptome analysis revealed that the gene encoding ODC (g4162) was significantly downregulated by 50.38% in the 1908-G20 generation compared with the 1908-G1 generation (Figure 6). Polyamines regulate the activity and subcellular localization of Rho GTPases through transglutaminase-catalyzed polyamination. Depletion of polyamines causes nuclear/perinuclear sequestration of Rac1 and RhoA and inhibits the activities of Rac1, RhoA, and Cdc42 [41].

In the Ras signaling pathway, all four genes encoding the guanine nucleotide exchange factor Son of sevenless (*Sos*) were downregulated in G20, among which g1915 exhibited a marked reduction of 53.7%. The highly expressed GTPase HRas gene g4350 was significantly downregulated by 41.52%, and the Ras-related C3 botulinum toxin substrate 1 (*Rac1*) gene g1 was also downregulated. These results demonstrate that the Ras signaling pathway in G20 mycelia was transcriptionally suppressed at multiple hierarchical levels. The coordinated decline in signal reception (*Sos*), transmission (*Ras*), and effector response (*Rac1*) likely impairs the regulation of the downstream actin cytoskeleton, consequently affecting hyphal tip growth and cell morphogenesis.

Taken together, these results indicate that polyamine metabolism and the Ras/Rho signaling pathway underwent a coordinated decline in G20 mycelia. The upstream signaling nodes *Sos*, *Ras*, and *Rac1* were consistently downregulated at the transcriptional level. In contrast, the downstream F-actin and actin beta/gamma 1 (*ACTB_G1*) genes displayed divergent expression patterns: g7538, g3392, and g8064 among the F-actin genes, as well as g11178 and g11225 among the *ACTB_G1* genes, were upregulated, whereas g7784 and g4430 (F-actin) and g11179 (*ACTB_G1*) were downregulated. This divergence suggests that the actin cytoskeleton in G20 mycelia has not yet undergone irreversible structural collapse but has entered a transitional state of abnormal remodeling. This is a key conclusion of the present study, highlighting that the cytoskeleton is actively but aberrantly reorganized under sustained upstream signaling attenuation. Given the sustained attenuation of upstream signaling inputs, eventual functional decline of the actin cytoskeleton may be anticipated.

### 3.10. RT-qPCR Verification of DEGs

To verify the reliability of the transcriptome sequencing results, four DEGs were selected to evaluate relative expression levels by RT-qPCR in 1908-G1 and 1908-G20 during liquid fermentation of *A. heimuer*. The validated DEGs included *ODC1* (g4162), *KRAS* (g4350), *DIB1* (g8624), and *UBP15* (g8705).

Before comparing expression patterns, the quality of the RT-qPCR assays was assessed. Melting curve analysis showed a single, sharp peak for all primer pairs (Figure S3), confirming amplification specificity. Ct values among biological replicates were highly consistent, and amplification curves were parallel, a pattern that is typical of uniform amplification efficiency. Primers were designed with stringent parameters (amplicon length 100–213 bp, Tm 58–61 °C, GC content 40–60%, no hairpin or self-complementary structures), yielding amplification efficiencies of 90–110% as validated by the manufacturer. All qPCR assays were performed using SGExcel FastSYBR Mixture (Sangon Biotech, Shanghai, China). Based on these combined observations, the amplification efficiency for all assays is inferred to fall within the 90–110% range, supporting the reliability of the qPCR quantification.

**Figure 7:**
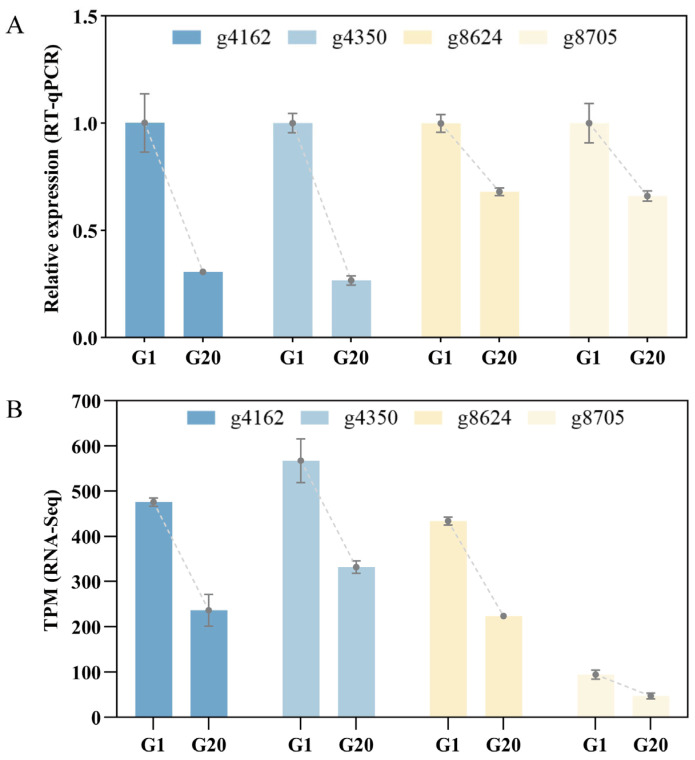
RT-qPCR results and transcriptome data of four DEGs. (**A**): RT-qPCR, (**B**): RNA-Seq.

The relative expression levels of the four DEGs detected by RT-qPCR were fully consistent in the direction of change with the transcriptome sequencing data (Figure 7). All four genes showed downregulation in both platforms under the tested conditions. This complete directional concordance confirms the reliability of the transcriptomic data.

To verify the reliability of the transcriptome sequencing results, a total of four DEGs were selected from the transcriptome data to evaluate relative expression levels through RT-qPCR in 1908-G1 and 1908-G20 during the liquid fermentation of *A. heimuer*. The validated DEGs included. The results demonstrated that the relative expression levels of these four DEGs detected by RT-qPCR in 1908-G1 and 1908-G20 were consistent with the transcriptome sequencing data (Figure 7), indicating that the transcriptome sequencing data are reliable.

## 4. Discussion

In recent years, the rapid development of *A. heimuer* industrial cultivation technologies has enabled large enterprises to master cultivation methods and establish their own technical systems [42]. Consequently, because of the substantial investment required for industrial production, the stability and quality of strains have become critically important. During subculturing, mycelial cells undergo a series of physiological activities, including chromosome replication, nuclear migration, and nuclear division [43,44,45]. These activities may potentially induce strain degeneration, thereby affecting production stability. Compared with multinucleate ascomycetes such as *Morchella* spp. [46], the binucleate basidiomycete *A. heimuer* is relatively stable during subculturing. However, although *A. heimuer* does not exhibit complete growth cessation at the 15th generation (G15), as observed in *Morchella* [47], many studies have still reported that subculturing can induce mycelial senescence or degeneration in *A. heimuer* [16,20,48].

In the preliminary stage of this study, ISSR clustering analysis was performed on successively subcultured strains of *A. heimuer* ‘HWS1908’ from generations G1 to G10 [49]. The results showed that the genetic similarity coefficients ranged from 0.94 to 1.00. At a similarity coefficient of 0.94, the strains were divided into two major groups: G1-G7 (I) and G8-G10 (II) (Figure S1). Beginning from G7, the mycelial tips started to narrow; although this alteration exerted some influence, it did not reach the threshold level (Figure S2). Therefore, subculturing was continued up to the 20th generation. Subsequently, systematic multi-level analyses, including phenotypic characterization, enzymatic activity assays, and transcriptomic profiling, were conducted on strains from generations G1, G4, G8, G12, G16, and G20. With increasing subculture generations, the mycelial growth rate gradually declined, becoming significantly reduced from G8 onward. At G20, the growth rate fell to 3.17 ± 0.08 mm/d, which was significantly lower than that of G1 (3.41 ± 0.03 mm/d, *p* < 0.05). This pattern is consistent with the subculturing-induced degenerative phenotypes reported in other edible fungi [19].

The activities of the antioxidant enzymes SOD, POD, and CAT all showed an overall increasing trend with successive subculturing, with SOD and POD peaking at G20; CAT exhibited a transient decrease at G8 but subsequently increased. These findings indicate that long-term subculturing induces chronic activation of the antioxidant system. Transcriptomic GO enrichment analysis revealed significant enrichment of terms such as “response to reactive oxygen species” and “cellular response to reactive oxygen species”, and KEGG analysis identified the peroxisome pathway as one of the most significantly enriched pathways. Together with the upregulation of antioxidant enzyme activities, these results strongly suggest that progressive oxidative stress acts as a primary trigger for mycelial degeneration [13]. The sustained upregulation of antioxidant enzymes likely represents an adaptive response to accumulating ROS.

As a typical white-rot fungus, *A. heimuer* secretes a variety of degrading enzymes to efficiently decompose lignocellulose and support vegetative growth [49,50]. In this study, cellulase and laccase activities decreased markedly starting from G8. Laccase activity declined continuously and fell to only 3.84 ± 0.09 U/g at G20. Cellulase activity hit the lowest level at G16 and picked up slightly at G20, but its value was still significantly lower than that at G1. Hemicellulase activity rose significantly by 22.73% at G20. Transcriptomic annotation identified 15 core lignocellulose-degrading CAZyme families that contained 83 DEGs in *A. heimuer* during continuous subculture, and more than half of these DEGs showed obviously downregulated expression. As important cellulase members, GH6 family genes participate in the hydrolysis of cellulose and β-1,4-glucan chains [31]. The prominent downregulation of these genes in strain 1908-G20 compared with 1908-G1 matched the reduced cellulase activity, and this finding verified weakened cellulose degradation capacity in degenerated mycelia. Lignin degradation mainly relies on lignin-modifying enzyme families including AA2 and AA6. The AA2 family consists of classic secreted heme peroxidases and plays a core role in lignin depolymerization. Intracellular AA6 proteins regulate the metabolism of aromatic intermediates and further accelerate lignin biodegradation [35]. All DEGs annotated to AA2 and AA6 families presented consistent downregulation in subcultured degenerated strains, which suggested obvious transcriptional inhibition of the lignin degradation system. Collectively, long-term asexual subculture triggered simultaneous repression of multiple functional CAZyme families relevant to cellulose and lignin degradation. Such a trend of reduced lignocellulolytic enzyme activities along with strain degeneration has also been documented in other edible fungi. In Lentinula edodes, laccase activity drops gradually with prolonged culture in degenerated strains [51]. Successive tissue isolation subculture can also lead to strain degeneration in *V. volvacea*. The mycelial colony diameter of T18 was 14.7% smaller than that of T0 with increasing subculture times, and the activities of exoglucanase, endoglucanase, laccase and hemicellulase decreased by over 30% [52].

In strain 1908-G20, long-term asexual subculture induced systematic transcriptional reprogramming of amino sugar and nucleotide sugar metabolism pathways, which directly resulted in an overall imbalance in cell wall chitin homeostasis. The fungal cell wall mainly consists of chitin and β-glucan, and intact chitin metabolism is essential for maintaining mycelial morphogenesis and stress resistance [53]. In this study, all eight genes encoding chitin synthases exhibited a downward expression trend, whereas the gene encoding UDP-*N*-acetylglucosamine pyrophosphorylase (g1931), a key upstream enzyme responsible for UDP-GlcNAc synthesis, was also downregulated (log_2_FC = −1.20). Since UDP-GlcNAc is the direct precursor substrate for chitin polymerization, insufficient synthesis restricts substrate availability for chitin synthases, and the overall reduction in chitin synthase expression further weakens chitin chain polymerization and elongation capacity [54]. Taken together, both precursor supply and polymerization/elongation pathways involved in chitin synthesis were significantly weakened in the degenerated strain 1908-G20. In contrast to the overall attenuation of the chitin synthesis pathway, the chitin degradation system exhibited a distinct differential regulatory pattern. Chitinases (EC 3.2.1.14) participate in cell wall remodeling, autolysis, and nutrient recycling during hyphal growth through cleavage of β-(1→4)-glycosidic bonds [55]. In this study, a core highly expressed chitinase gene, g6136, was markedly downregulated by 59.76% (log_2_FC = −1.40), whereas another highly expressed chitinase gene, g4406, was upregulated by 27.80% and remained highly expressed. This divergent expression pattern within the chitinase family suggests that the overall chitinolytic activity in strain 1908-G20 was not completely abolished; rather, a certain capacity for cell wall remodeling and turnover was retained. Previous studies demonstrated functional differentiation among chitinase family members, which are phylogenetically classified into three major groups and can be further subdivided into several subgroups [56]. Therefore, it is plausible that g6136 and g4406 perform distinct functional roles within the chitin degradation network: the downregulation of g6136 likely reflects attenuation of growth-associated chitinase activity, whereas the upregulation of g4406 may represent a compensatory mechanism to maintain basal cell wall metabolism under degenerative stress. In summary, suppression of chitin synthesis together with maintenance of chitin degradation in strain 1908-G20 suggests a progressive loss of the capacity to sustain normal chitin homeostasis in degenerated mycelia, thereby affecting apical dominance and branching patterns during hyphal growth. This observation is consistent with the tapered hyphal tips observed in the degenerated strain [20].

The actin cytoskeleton functions as the executive machinery for hyphal tip growth and morphogenetic maintenance in filamentous fungi, and its dynamic organization is tightly regulated by Ras and Rho family small GTPases [57]. This study provides evidence of multilayered transcriptional suppression along this regulatory axis during subculture-induced degeneration of *A. heimuer*. At the signal reception level, gene g1915 encoding the guanine nucleotide exchange factor *Sos* exhibited a pronounced downregulation of 53.7%. Since *Sos* functions as a key positive regulator that catalyzes the conversion of Ras from an inactive GDP-bound state to an active GTP-bound state, its substantial downregulation likely weakens the activation capacity of Ras signaling. In *Aspergillus nidulans*, the Ras GAP protein GapA has been demonstrated to be essential for apical polarization of the actin cytoskeleton [58]. Simultaneously, the expression of both *Ras* (g4350) and *Rac1* (g1) was also downregulated in this study, forming a coordinated suppression cascade extending from upstream activators to downstream effectors. This multilayered attenuation would likely reduce regulatory control over the actin cytoskeleton, thereby impairing hyphal tip growth and cell morphogenesis. In *Penicillium expansum*, deletion of *rasA* and *rasB* similarly causes growth defects and hyphal malformation [59], which is highly consistent with our observations. More importantly, this study revealed a close functional coupling between polyamine metabolism and the Ras/Rho signaling pathway, both of which underwent coordinated decline during degeneration. Polyamines fulfill multiple essential functions in fungal hyphal growth and stress responses. In the ectomycorrhizal fungus *Paxillus involutus*, putrescine content declines markedly with increasing hyphal age [39]. In the present study, the gene encoding ODC (g4162), the rate-limiting enzyme in polyamine biosynthesis, was significantly downregulated by 50.38% in G20, suggesting that polyamine synthesis may be restricted. Polyamines regulate the activity and subcellular localization of Rho GTPases through transglutaminase-catalyzed polyamination. Depletion of polyamines causes abnormal nuclear/perinuclear sequestration of *Rac1* and *RhoA*, thereby inhibiting their activities [41]. Therefore, downregulation of ODC not only reflects an intrinsic decline in polyamine metabolism but may also further aggravate actin cytoskeleton dysregulation through weakening the activities of *Rac1* and other Rho GTPases. Notably, at the downstream executive level, F-actin and *ACTB_G1* genes exhibited divergent expression patterns, with some members upregulated and others downregulated. This divergence indicates that the actin cytoskeleton in G20 mycelia has not yet undergone irreversible structural collapse but has instead likely entered a transitional state of abnormal remodeling. Previous studies have shown that although the actin cortex may persist following polyamine depletion, its capacity for dynamic reorganization is severely impaired [60]. Given the sustained attenuation of upstream signaling inputs, eventual functional decline of the actin cytoskeleton is a plausible outcome. It has also been reported in *A. heimuer* that mycelia become more slender and sparsely branched following successive subculture [48]. This morphological alteration closely corresponds to the downregulation of the Arp2/3 complex observed in this study, indicating reduced branched actin nucleation capacity, as well as compositional remodeling of F-actin.

From an evolutionary standpoint, basidiomycetes such as *Auricularia heimuer* maintain stable genomes due to their binucleate heterokaryotic state. Repeated asexual subculture can break the balanced coexistence of two different nuclei. This change further causes heterokaryosis loss and subsequent strain degeneration. The gradual degenerative phenotype found in our experiment is also comparable to replicative senescence in model fungi. Accumulated oxidative injury and continuous telomere shortening are core driving factors for physiological aging in such fungal species. We still cannot define the exact nature of subculture-triggered degeneration. This degenerative trait may originate from a protective response against accumulated mutations or replicative exhaustion. It may also arise from the disrupted balance between two cell nuclei. Cross-generational comparative genome and karyotype testing will help researchers tell apart these three potential causes.

We built a complete molecular regulatory model for *A. heimuer* subculture degeneration based on combined physiological and transcriptome results. Long-term successive asexual subculture induces persistent oxidative stress. Elevated antioxidant enzyme activities, enriched reactive oxygen species-related GO entries and activated peroxisome pathway all support this conclusion. The sustained oxidative stress works as the core initiating factor for three major molecular changes. First, multiple lignocellulose degrading CAZyme gene families including GH6, AA2 and AA6 are transcriptionally inhibited. This transcriptional inhibition directly lowers the activity of cellulase and laccase. Second, key genes participating in chitin synthesis such as chitin synthases and UDP-GlcNAc pyrophosphorylase show decreased expression levels. The weakened chitin synthesis further damages structural integrity of the fungal cell wall. Third, the polyamine synthesis pathway marked by reduced ODC expression and the downstream Ras/Rho signal cascade covering Sos, Ras and Rac1 are synchronously suppressed. Such coordinated inhibition disturbs normal actin cytoskeleton remodeling and restrains apical extension of fungal hyphae. Several chitinase genes like g4406 keep partial enzymatic activity during strain degeneration. This phenomenon is regarded as an adaptive compensatory strategy for mycelial survival. All the above molecular abnormalities jointly lead to visible degenerative traits. These typical traits contain slowed vegetative growth, weakened substrate decomposition capacity and abnormal mycelial morphology. The G20 generation stands for a vital critical turning point. Degenerative symptoms develop sharply after this generation, and further continuous subculture will probably bring permanent irreversible functional damage.

The significantly downregulated genes including ODC, Sos, Ras and Rac1 can be used as candidate molecular biomarkers for early diagnosis of strain degradation in factory production labs. For example, a reduction in ODC or Ras transcript levels by more than 40% relative to G1 could serve as an actionable threshold for strain replacement. Cultivation technicians can regularly detect the transcription abundance of these target genes. They can replace or rejuvenate degraded strains in advance once the tested batches are close to the G20 critical threshold. This finding bridges basic molecular research and practical edible fungus production. It can effectively cut economic losses and lay a foundation for unified industrial subculture specification in the domestic black fungus industry. However, all experimental data in this work are obtained from one single strain under fixed in vitro culture environments. Researchers need to verify the accuracy and stability of these candidate biomarkers on various commercial strains and practical production conditions before formal quality control application. Transcriptome analysis clarifies the underlying degeneration mechanism at transcriptional level. Related results still need supplementary verification from proteome and metabolome detection in follow-up experiments. Subsequent multi-omics research will confirm whether differential gene expression can induce corresponding variation in protein content and intracellular metabolic flow. Standard laboratory culture environments cannot fully simulate diverse adverse stresses in large-scale factory cultivation. More field studies are essential to explore real degeneration rules under industrial production settings.

## 5. Conclusions

In this study, we adopted the Illumina high-throughput transcriptome sequencing technique. We carried out reference-based transcriptome analysis with the available *A. heimuer* genome (GCA_050574805.1). Our main purpose was to screen differentially expressed genes between the G1 and G20 subculture generations of strain ‘HWS1908’. We identified a total of 2643 DEGs in this comparison group. These genes are functionally linked to fungal growth and development. They also participate in oxidative stress response, cell wall maintenance and substrate decomposition. Under our present culture conditions, the G20 generation serves as a critical threshold for strain degeneration. This threshold is reflected by slower mycelial growth speed and obviously decreased lignocellulolytic enzyme activities. It is also accompanied by transcriptional downregulation of core functional signaling pathways. Cumulative evidence from enriched ROS-related GO entries, elevated antioxidant enzyme activities and activated peroxisome pathway supports that oxidative stress is the core initiating factor of strain degeneration. Continuous oxidative damage further suppresses transcription of lignocellulose degrading enzyme genes and chitin synthesis-related genes. It also inhibits the polyamine-dependent Ras/Rho signaling pathway at transcriptional level. All these molecular changes eventually induce physiological recession and mycelial degeneration. We suggest controlling the subculture number below 20 generations for the daily preservation of this industrial strain. This threshold may vary under different culture conditions or for other strains, and should be validated before industrial application. Further verification is still needed under diversified production settings. Subsequent functional verification is required for those hub genes screened from transcriptome data. Researchers can adopt gene overexpression or gene knockdown technology to clarify their exact biological roles during degeneration. Moreover, systematic fruiting experiments with serially subcultured strains should be arranged. These trials can monitor the variation in fruiting body morphology and total yield. The relevant data will help us explore how successive subculturation influences the reproductive development of *A. heimuer*.

## Data Availability

The data presented in this study are openly available in NCBI at BioProject ID: PRJNA1467312, BioSample accessions: SAMN60240833, SAMN60240834.

## Supplementary Materials

The following supporting information can be downloaded at: https://www.mdpi.com/article/10.3390/jof12060437/s1, Table S1. The primers used in this study. Table S2. The top 30 DEGs on GO, sorted by Qvalue. Table S3. The expression patterns of DEGs related to GH18 family. Table S4. The DEGs of CAZyme families involved in the wood degradation. Figure S1. The result of UPGMA clustering analysis [20]. Figure S2. The mycelium and tip diameter of strain ‘HWS1908’ after 10 subcultures [20]. Figure S3. The melting curve of four genes.
