# Supplementary material for: Analysis of the Subculture Effect on the Auricularia heimuer Strain ‘HWS1908’ Based on Transcriptome
Source: J Fungi (Basel). 2026 Jun 15;12(6):437. doi: 10.3390/jof12060437 (PMC13301363; doi:10.3390/jof12060437)
Supplement: Supplementary file 1 [file jof-12-00437-s001.zip › jof-4347223-supplementary.pdf]

Table S1. The primers used in this study.

Gene	F(5'-3')	R(5'-3')
APRTase	CGCTCGCAGGGTCTCAAGAATG	GCCGTGGAAGTTCGTCAAGCA
g4162	GCGAGGTCGGCTATCAGTTTAC	CGCTCATTCTTCGGGAAATAGTA
g4350	GACGATTCAGTTCATCCAGAGC	AACGAGTTGCGGGAGGTAA
g8624	TTCGGCCACGACTGGGAC	TTGGCATGGCCCAGTT
g8705	GAGGACCAGGCTCCGCCG	CCACTCCAGCTCGAGTTTCG

Table S2. The top 30 DEGs on GO, sorted by Qvalue.

Sort	GO.ID	Term	Ontology	Significant	Pvalue	Qvalue
1	GO:0042026	protein refolding	biological process	11	2.4e-06	0.0140976
2	GO:0030684	preribosome	cellular component	39	3.1e-05	0.032581
3	GO:0034605	cellular response to heat	biological process	20	3.1e-05	0.091047
4	GO:0009408	response to heat	biological process	21	6.7e-05	0.131186
5	GO:0000302	response to reactive oxygen species	biological process	15	0.0001	0.14685
6	GO:0034614	cellular response to reactive oxygen species	biological process	13	0.00015	0.17622
7	GO:0006364	rRNA processing	biological process	46	0.00019	0.17622
8	GO:0009266	response to temperature stimulus	biological process	21	0.00029	0.17622

9	GO:0010446	response to alkaline pH	biological process	5	0.0003	0.17622
10	GO:0071469	cellular response to alkaline pH	biological process	5	0.0003	0.17622
11	GO:1990481	mRNA pseudouridine synthesis	biological process	5	0.0003	0.17622
12	GO:0042744	hydrogen peroxide catabolic process	biological process	4	0.0004	0.20559
13	GO:0006457	protein folding	biological process	27	0.00042	0.20559
14	GO:0016072	rRNA metabolic process	biological process	54	0.00053	0.211464
15	GO:0009628	response to abiotic stimulus	biological process	43	0.00054	0.211464
16	GO:0061077	chaperone-mediated protein folding	biological process	13	0.00061	0.2239462 5
17	GO:0030686	90S preribosome	cellular component	21	0.00084	0.33632
18	GO:0005576	extracellular region	cellular component	26	0.00139	0.33632
19	GO:0033573	high-affinity iron permease complex	cellular component	4	0.0016	0.33632
20	GO:1905862	ferroxidase complex	cellular component	4	0.0016	0.33632
21	GO:0008152	metabolic process	biological process	434	0.00142	0.4381025
22	GO:0005975	carbohydrate metabolic process	biological process	44	0.00157	0.4381025
23	GO:0043934	sporulation	biological process	30	0.00177	0.4381025
24	GO:0006048	UDP-N-acetylglucos amine biosynthetic process	biological process	4	0.00179	0.4381025
25	GO:0033215	reductive iron assimilation	biological process	4	0.00179	0.4381025
26	GO:0042743	hydrogen peroxide metabolic process	biological process	4	0.00179	0.4381025

27	GO:0004322	ferroxidase activity	molecular function	4	0.00203	0.47096
28	GO:0016724	oxidoreductase activity, acting on metal ions, oxygen as acceptor	molecular function	4	0.00203	0.47096
29	GO:0032993	protein-DNA complex	cellular component	17	0.00396	0.500276
30	GO:0005657	replication fork	cellular component	18	0.00455	0.500276

Table S3. The expression patterns of DEGs related to GH18 family.

ID	log_2Fold	pValue	qValue	result	TPM (G20-1)	TPM (G1-1)	CAZy
g9108	-1.640947978	7.9288767894846e-13	4.84629424961433e-12	down	2.07	6.14	CBX99002.1|GH18|
g9475	-1.088629243	3.08615279236469e-18	2.8864605528587403e-17	down	173.84	346.86	CBX99002.1|GH18|
g9527	1.40808307792553	6.79952444782982e-43	2.3189334682077e-41	up	50.18	17.79	QKX63840.1|CBM18|GH18|
g9747	1.63603517599074	9.75905581434169e-16	7.6652236790083e-15	up	7.58	2.28	AIT18900.1|GH18|
g9895	-1.266154716	9.55377887399533e-10	4.2741354460619404e-09	down	1.41	3.18	SJX66486.1|GH18|
g10440	4.81039884171753	1.93966897901733e-100	2.81754879100221e-98	up	112.84	3.74	ACV60538.1|GH18|3.2.1.96
g11370	1.1904308898996	2.0435912569593e-11	1.06961275486579e-10	up	76.47	31.42	AIT18866.1|GH18|
g989	-2.523636919	8.243240006920801e-32	1.6985982823367797e-30	down	1.20	6.54	CCA69604.1|CBM5|GH18|
g1407	-1.309251795	5.839375277768519e-16	4.6612001030003e-15	down	59.81	138.88	AUB25287.1|GH18|
g13028	-1.058605005	4.76552055246152e-16	3.82737985121244e-15	down	81.14	158.80	VWO96832.1|GH18|
g4381	3.19321634885731	6.55550704306569e-54	3.2545188130257802e-52	up	1006.26	103.31	VWO96832.1|GH18|
g4700	1.49308923584221	1.7667475742916602e-23	2.33567999540626e-22	up	68.60	22.90	CBX99002.1|GH18|

g5127	-2.767526675	7.91266767593186e-33	1.71140529182012e-31	down	137.96	891.64	AQT80722.1|GH18|
g6136	-1.401789118	4.41490659773818e-12	2.47951269828102e-11	down	480.87	1194.97	CCA69604.1|CBM5|GH18|
g6769	-2.257954814	3.99674903585061e-74	3.07357837619727e-72	down	11.56	52.01	QKD55908.1|GH18|
g6785	-1.080483232	9.45140845848654e-30	1.74851056482001e-28	down	66.97	132.99	CBX99002.1|GH18|
g7586	2.14350230804483	1.5957378816770794e-11	8.46247591267202e-11	up	12.91	2.69	VWO96832.1|GH18|

Table S4. The DEGs of CAZyme families involved in the wood degradation.

Substate	gene id	log_2Fold	result	TPM (1908-G20-1)	TPM (1908-G1-1)	CAZy
Crystalline cellulose	g10874	4.12	up	725.25	39.19	QLI66438.1|CBM1|
	g11239	2.09	up	4.57	1.01	CAP68330.1|CBM1|
	g12167	1.30	up	3135.30	1200.96	AFR33047.1|CBM1|GH10|
	g144	-2.06	down	30.38	119.52	AGV52632.1|CBM1|GH5_7|
	g13505	-1.61	down	0.50	1.44	ATZ51220.1|CBM1|CE16|
	g13517	2.89	up	5.15	0.64	CCA73365.1|CBM1|CE16|
	g3527	-1.16	down	27.83	58.63	EGX47404.1|CBM1|CE15|
	g3634	5.90	up	0.51	0.00	QLI66438.1|CBM1|
	g4964	1.47	up	0.35	0.12	AEO68387.1|CBM1|CE16|
	g5925	4.85	up	1097.76	35.52	QLI66438.1|CBM1|
	g7367	-1.12	down	7.15	14.63	CDJ79827.1|CBM1|GH74|
	g7550	1.33	up	5.02	1.88	VWO94258.1|CBM1|GH5_5|
	g835	-2.43	down	8.10	40.93	AWK40335.1|GH6|
	g12828	-2.90	down	169.83	1194.45	AXG45674.1|GH6|
	g1720	-1.63	down	94.80	276.63	AWK40335.1|GH6|
	g10727	-1.71	down	2.06	6.31	CCA67659.1|AA9|CBM1|
	g176	2.08	up	0.37	0.08	VBB84479.1|AA9|
	g5303	1.53	up	71.41	23.06	QKX62378.1|AA9|
	g5701	-1.26	down	11.68	26.21	CAG27578.1|AA9|
	g8625	1.12	up	51.97	22.33	SMR52966.1|CE16|
	g9047	-1.15	down	52.32	109.82	SMR52966.1|CE16|
	g1905	-1.52	down	19.06	51.62	QGI94678.1|CE16|
	g2416	-3.88	down	2.50	34.67	VWO98374.1|CE16|
	g2615	-2.79	down	11.57	75.22	SMY24715.1|CE16|

	g3665	-1.91	down	0.61	2.17	VWO98374.1|CE16|
	g3666	1.75	up	6.30	1.72	VWO98374.1|CE16|
	g3669	1.61	up	2.80	0.85	VWO98374.1|CE16|
	g3670	1.19	up	11.60	4.78	VWO98374.1|CE16|
	g3677	-1.00	down	8.47	15.89	VWO98374.1|CE16|
	g6347	1.00	up	32.23	15.13	CCA70820.1|CE16|
	g7869	-1.73	down	122.06	381.17	SMY24715.1|CE16|
	g5266	1.10	up	26.87	11.68	AII51353.1|CE1|
	g10616	2.27	up	17.72	3.46	ARA85284.1|CE12|
	g10634	2.33	up	524.41	97.36	ARA85284.1|CE12|
	g1257	-1.25	down	0.39	0.88	CAP67203.1|CE12|
	g1277	-2.82	down	1.23	8.32	CAP67203.1|CE12|
	g336	1.04	up	5.75	2.64	CBX90574.1|CE15|
Lignin	g10628	2.09	up	42.18	9.29	QKX61286.1|AA1|
	g396	-1.07	down	185.64	366.20	VUW72999.1|AA1|
	g4659	-1.15	down	2.25	4.70	SMR45619.1|AA1|
	g7075	1.02	up	5.66	2.62	VUW72999.1|AA1|
	g1988	1.19	up	61.04	24.92	AHZ58332.1|AA1_2|
	g9609	1.18	up	15.77	6.55	AHZ58328.1|AA1_3|
	g119	-1.07	down	0.46	0.92	AHZ58330.1|AA1_3|
	g5483	3.73	up	0.49	0.03	AHZ58333.1|AA1_3|
	g9620	-2.59	down	50.71	288.46	AZJ17948.1|AA2|
	g9738	-1.31	down	10.03	23.39	CAD56164.1|AA2|
	g11116	-1.00	down	5.79	10.95	AZJ17944.1|AA2|
	g6061	-2.21	down	29.42	127.28	QLI73446.1|AA2|
	g9039	-1.12	down	149.17	304.98	QKX58234.1|AA3_2|
	g9732	-1.58	down	5.77	16.18	QKD61481.1|AA3_2|
	g9756	2.55	up	6.71	1.07	QBZ61175.1|AA3_2|
	g10077	1.34	up	17.34	6.38	QBZ61175.1|AA3_2|
	g10087	-1.87	down	120.06	412.91	QHB50190.1|AA3_2|
	g10639	2.89	up	15.65	1.98	EGX43942.1|AA3_2|CBM1|
	g11376	-1.16	down	5.68	11.92	EAA61740.1|AA3_2|
	g11501	-2.80	down	9.17	60.41	QBZ61175.1|AA3_2|
	g842	-2.76	down	3.68	23.40	SJX61802.1|AA3_2|
	g1113	-3.30	down	16.82	154.81	CCA75795.1|AA3_2|
	g12854	-3.44	down	20.60	210.17	QKX58234.1|AA3_2|
	g13155	1.74	up	5.21	1.45	EGX43942.1|AA3_2|CBM1|
	g2094	1.37	up	11.92	4.29	EGX43942.1|AA3_2|CBM1|

g3336	-1.28	down	69.00	158.00	SMQ49497.1|AA3_2|
g4467	-1.17	down	26.18	55.27	QDS77604.1|AA3_2|
g5257	-1.39	down	5.00	12.29	ALJ82901.1|AA3_2|
g8538	-1.02	down	6.72	12.88	SAM80724.1|AA3_2|
g9327	2.01	up	253.01	58.45	ABI14440.1|AA3_3|
g7195	-3.18	down	0.77	6.62	VWO96530.1|AA3_3|
g1702	-6.66	down	1.42	134.80	ALJ82907.1|AA3_4|
g8707	-2.17	down	55.97	236.40	ABD61574.1|AA5_1|
g9781	-2.68	down	1.95	11.79	QIW95400.1|AA5_1|
g13143	-1.31	down	276.76	647.12	ABD61573.1|AA5_1|
g1930	-1.86	down	4.50	15.41	QIW95400.1|AA5_1|
g3716	-3.08	down	0.76	6.03	ABD61577.1|AA5_1|
g5134	-3.29	down	52.13	479.79	QIW95400.1|AA5_1|
g5838	-1.85	down	28.49	96.29	ABD61573.1|AA5_1|
g12090	-2.32	down	22.63	105.94	AAQ24589.1|AA6|
g1231	-2.35	down	195.65	941.71	VWP01316.1|AA6|
g1668	-2.75	down	24.12	152.53	VWP01316.1|AA6|
g5916	-1.40	down	4.62	11.42	QHO20297.1|AA6|
g3643	2.95	up	15.33	1.89	CAI94231.1|AA7|
g4137	-2.14	down	48.48	200.76	CCA70426.1|AA7|
g4493	1.66	up	51.32	15.10	CCA70426.1|AA7|

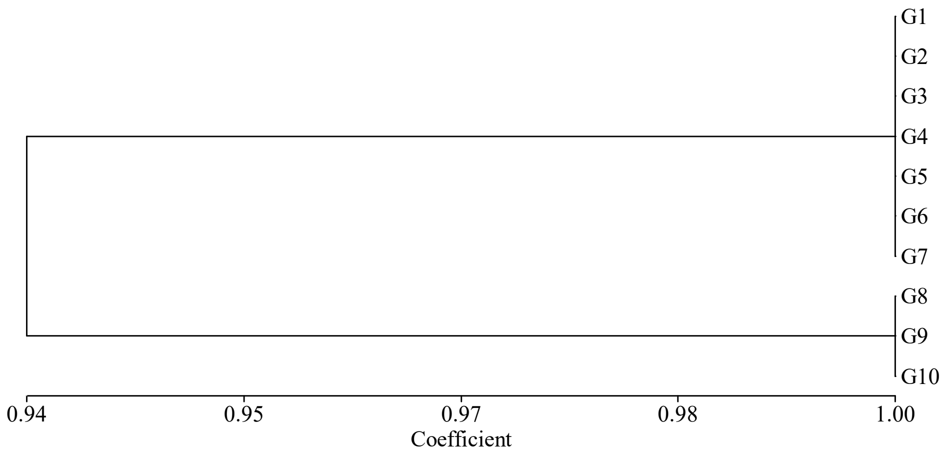

Figure S1. The result of UPGMA clustering analysis [20].

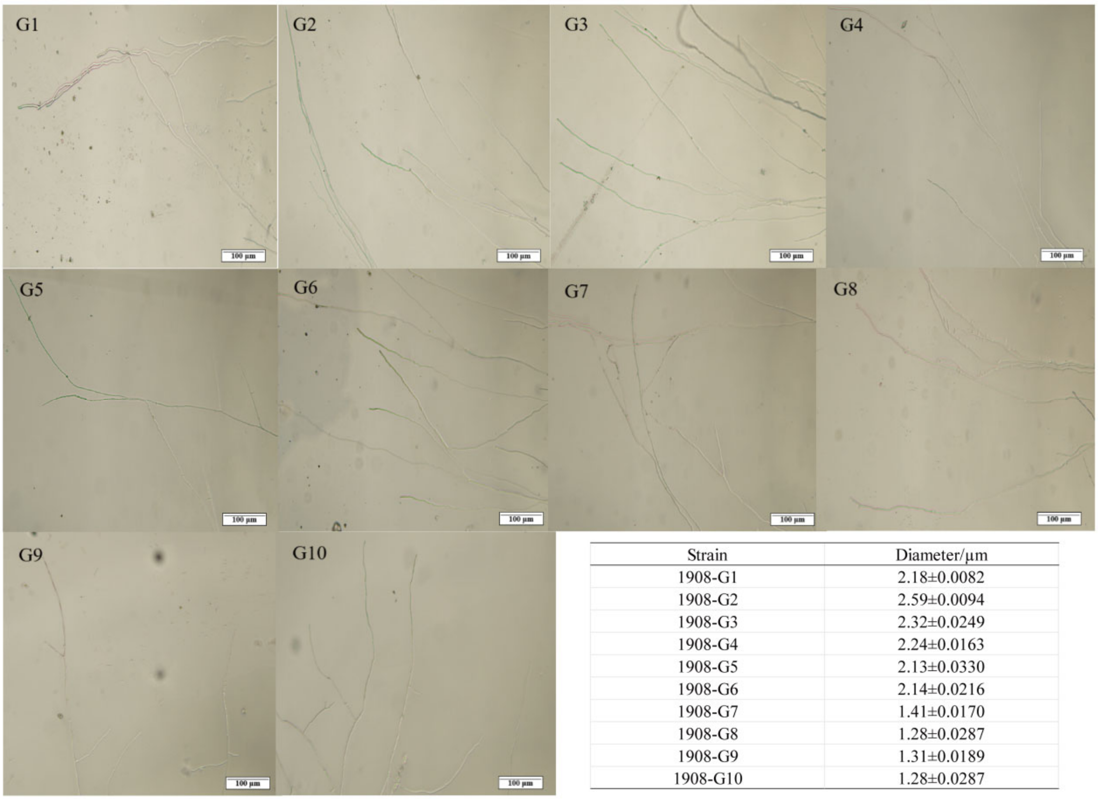

Strain	Diameter/μm
1908-G1	2.18±0.0082
1908-G2	2.59±0.0094
1908-G3	2.32±0.0249
1908-G4	2.24±0.0163
1908-G5	2.13±0.0330
1908-G6	2.14±0.0216
1908-G7	1.41±0.0170
1908-G8	1.28±0.0287
1908-G9	1.31±0.0189
1908-G10	1.28±0.0287

Figure S2. The mycelium and tip diameter of strain 'HWS1908' after 10 subcultures [20].

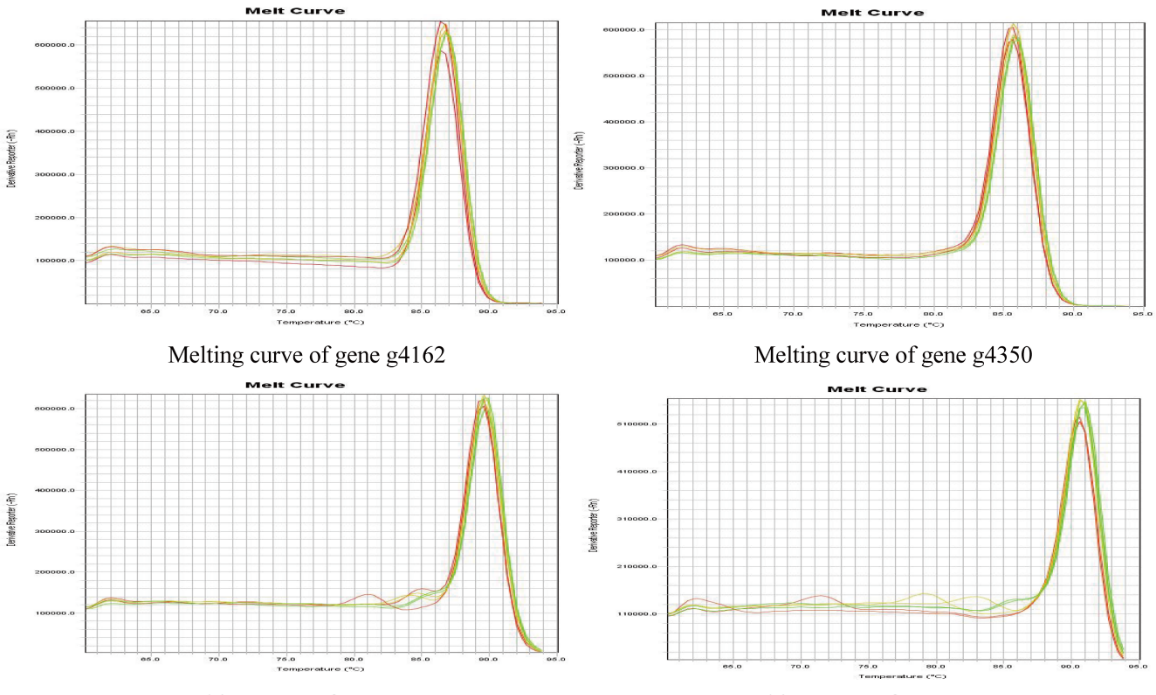

Melting curve of gene g4162

Melting curve of gene g4350

Melting curve of gene g6715

Melting curve of gene g4406

Figure S3. The melting curve of four genes.
